# Current status and future directions in the management of chronic hepatitis C

**DOI:** 10.1186/1743-422X-9-57

**Published:** 2012-03-02

**Authors:** Wosen Aman, Shaymaa Mousa, Gamal Shiha, Shaker A Mousa

**Affiliations:** 1 The Pharmaceutical Research Institute, Albany College of Pharmacy and Health Sciences, Rensselaer, NY, USA; 2 Faculty of Medicine, Almansoura University, Almansoura, Egypt

**Keywords:** Hepatitis C, Fibrosis, Cirrhosis, Hepatic carcinoma, Prevention, Treatment, Antiviral

## Abstract

Hepatitis C virus (HCV) is endemic worldwide, and it causes cirrhosis and other complications that often lead to death; nevertheless, our knowledge of the disease and its mechanisms is limited. HCV is most common in underdeveloped nations, including many in Africa and Asia. The virus is usually transmitted by parenteral routes, but sexual, perinatal, and other types of transfer have been known to occur. Approximately 80% of individuals who contract hepatitis C develop a chronic infection, and very few are able to spontaneously clear the virus. Because hepatitis C is asymptomatic in the majority of patients, the presence of HCV RNA in the serum is the best diagnostic tool. Although serious complications from hepatitis C may not occur for 20 years, 1/5 of chronic patients eventually develop life - threatening cirrhosis. More research is needed on the different therapy options for the disease, and many factors, most importantly the genotype of the virus, must be taken into account before beginning any treatment. As there is no vaccine against HCV at present, the most effective and recommended therapy is pegylated-interferon-α-2a plus ribavirin. While interferon is marginally effective as a monotherapy, both adding the moiety and combining it with ribavirin have been shown to dramatically increase its potency. While there are numerous alternative and complementary medicines available for patients with hepatitis C, their efficacy is questionable. Currently, research is being done to investigate other possible treatments for hepatitis C, and progress is being made to develop a vaccine against HCV, despite the many challenges the virus presents. Until such a vaccination is available, prevention and control methods are important in containing and impeding the spread of the virus and mitigating its deleterious effects on the health of people and communities worldwide.

## Introduction

**Figure 1:**
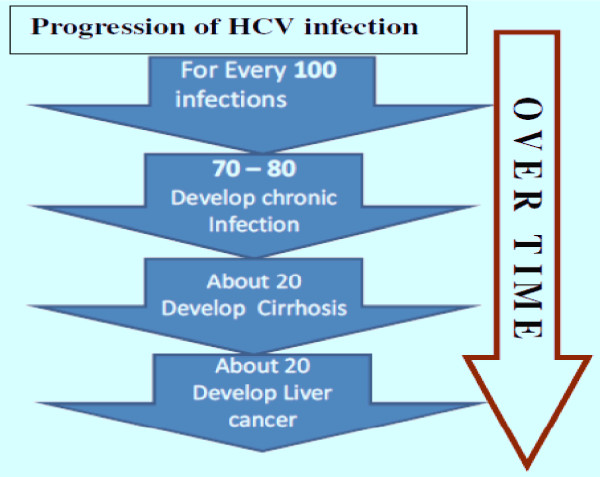
**Progression of HCV Infection**.

The hepatitis C virus (HCV) infects up to 170 million people throughout the world, causing chronic liver disease, inflammation, and long-term complications [1-3]. HCV is a member of the Flaviviridae family, has single-stranded RNA, and is relatively small (55 nm - 65 nm) [1-4]. In 1989, it was determined that HCV is responsible for most transfusion-associated non-A and non-B hepatitis infections [2,5]. Eleven different genotypes of the virus have been identified, each with various subtypes and strains, but 60% of HCV infections are caused by type 1a and type 1b [1,5]. The majority of those exposed to HCV become chronic carriers of the virus; only 20% - 30% are expected to be free of the virus within six months of infection [1,3,6]. Nearly 20% of chronic carriers develop cirrhosis, while another 20% develop liver cancer [1], (Figure 1). HCV is also the leading cause of hepatocellular carcinoma and cirrhosis [5].

### Acute and chronic hepatitis C

The severity of hepatitis C ranges from mild, short-term symptoms to complicated, life-long liver disease that can result in death. Whether the infection is acute or becomes chronic depends on the duration of the virus' existence in the patient's body.

Acute hepatitis C is a short-term infection of the virus that occurs during the first six months after exposure to HCV. The acute stage of the disease is characterized by elevated serum alanine aminotransferase (ALT) levels and jaundice, which appears in about 25% of cases [1,5,7]. About 80% of acute hepatitis C cases are asymptomatic, and are therefore difficult to diagnose [5,8]. However, if a patient's symptoms and/or lifestyle are suggestive of an HCV infection, they should be tested for the disease. The presence of HCV RNA in the serum is the most reliable way to diagnose hepatitis C, but the presence of anti-HCV antibodies in the serum or an elevated serum ALT level (a concentration at least ten times the upper limit of the normal range is necessary for a diagnosis) can also indicate an HCV infection [1,3,5,7]. The remaining 20% of cases are symptomatic, and are characterized by poor appetite, abdominal pain in the right upper quadrant, joint and muscle pains, nausea, vomiting, and fever [1,3,5,7]. However, because these symptoms are common and inconclusive, symptomatic hepatitis C should be diagnosed by the same tests used to confirm an asymptomatic infection.

An acute hepatitis C infection becomes a chronic disease if the individual is unable to clear the virus from their system within six months of infection without any therapeutic intervention; approximately 70% - 80% of those with an acute infection develop chronic hepatitis C [1,5,6]. Chronic hepatitis C is officially diagnosed by the presence of anti-HCV and the elevation of serum ALT levels for more than six months [3]. Even in the chronic stage, however, most infected persons show no symptoms until serious complications arise, although this often does not occur until 20 years after infection [1,3,5]. As such complications -- including hepato-cellular carcinoma, liver failure, and cirrhosis -- develop as the infection progresses, more prominent symptoms such as swelling of the abdomen, fatigue, dark urine, fluid retention, jaundice, ascites, muscle weakness, itching, and weight loss are observed [3,5]. Cirrhosis is perhaps the most well known consequence of chronic hepatitis C, and occurs in about 20% of patients [1,5]. About 20% - 25% of patients who do develop cirrhosis as a result of HCV die from liver failure [5].

### Treatment of hepatitis C

The goal of hepatitis C treatment is to prevent the development of chronic infection, increase the patient's quality of life, and prevent morbidity and mortality [9]. However, the type and duration of therapy a patient receives depends on a number of factors, including age, the genotype of the virus, and any previous conditions the patient may have. For example, individuals with decompensated cirrhosis, severe uncontrolled psychiatric disorders, advanced cardiovascular or cerebrovascular disease, creatinine clearance of less than 50 mL/min, or women who are pregnant should not receive therapy [3,9,10]. Additionally, more research is needed on the proper treatment protocol for children, which is likely different than that for adults [11,12].

So far, 11 HCV genotypes have been discovered, but only genotypes 1, 2 and 3 are distributed worldwide, and about 60% of infections are due to type 1a and 1b [1,5]. The genotype of the virus plays a substantial role in determining the duration and type of treatment the patient receives (viral load of serum is the other main factor to consider); thus it is necessary to confirm the genotype of a specific infection in order to plan an appropriate therapeutic strategy [2,3,5]. Although all genotypes exhibit relatively equal virulence, they differ in their response to a given medication [1-5]. In general, any treatment involving Interferon (IFN) is more effective against HCV genotype 2 and 3 than against genotype 1, which is less responsive to therapy [1-5]. Thus, a 48-week course of treatment is recommended for genotype 1, whereas a 24-week therapy is sufficient for genotypes 2 and 3 [2,3,5].

### Non-pharmacologic treatments

After a patient is diagnosed with hepatitis C, lifestyle changes must be made in order to limit and control further health problems. These non-pharmacologic treatments include engaging in regular physical exercise, adopting healthy eating habits, and maintaining a healthy weight. The latter is especially important, because it is believed that obesity in HCV patients plays a significant role in the progression of fibrosis [13,14]. Also, patients should avoid drinking alcohol because there is no safe minimum limit of consumption, and alcohol is one of the major risk factors for liver disease and exacerbates the negative effects of hepatitis C [3,15,16]. Additionally, hepatitis C patients are advised not to smoke because smoking may contribute to the progression of the disease and increase the likelihood of developing hepatocellular carcinoma [9,17].

### Pharmacologic treatments

Most cases (72%-98%) of symptomatic acute hepatitis C are curable if diagnosed early [18]. However, because some patients with symptomatic HCV may spontaneously clear the virus from their systems within 12 weeks of infection, therapy should not begin until after this time period has elapsed in order to avoid unnecessary treatment and medical costs [8,18-20]. On the other hand, because relatively few individuals with asymptomatic acute hepatitis C are able to spontaneously clear the virus, in such cases the appropriate treatment should be initiated as soon as possible [18,20]. However, the rate of spontaneous clearance is debatable; some sources indicate that around 31% of symptomatic cases and about 18% of asymptomatic cases are naturally resolved without treatment [18], while others suggest higher figures. A study by Geralch et al., for instance, showed that permanent and spontaneous clearance of the virus was observed in more than 50% of untreated patients with acute symptomatic HCV within the first three to four months after infection [20]. Those who remained infected after that time period were treated with either IFN-monotherapy, or with IFN plus ribavirin, and about 80% of those patients showed a sustained biochemical and virological response. Initiating treatment in patients with acute symptomatic HCV three months after infection led to viral clearance in 91% of the patients as a result of both spontaneous clearance and anti-viral treatment [20]. Regardless of the exact figures, there is a general consensus that asymptomatic patients should begin receiving treatment immediately, while symptomatic patients may benefit from postponing therapy for several months.

### Pegylated interferon-alpha monotherapy

The covalent attachment of a polyethylene glycol (peg) moiety to IFN-α reduces the clearance and degradation of the drug, thus sustaining a high plasma concentration and prolonging the half-life of the compound, respectively [2,3]. There are currently two versions of this compound known as pegylated interferon (peg-IFN) being used to treat hepatitis C: peg-IFN-α-2a and peg-IFN-α-2b. Common side effects of peg-IFN-α treatment include headache, fatigue, depression, insomnia, fever, nausea, myalgia, weakness, hematologic disorder, and injection site reaction [21]. Studies show that a once weekly administration of peg-IFN-α-2a is more efficient than a regimen of IFN-α-2a [22-24]. A comparison between the clinical effects of a regimen of peg-IFN-α-2a and a regimen of IFN-α-2a was made in the study by Zeuzem et al. (Table 1). The results show a profound improvement in both virology and biochemical responses after 48 and 72 weeks [22].

**Table 1:** A summary of the effects of Peg-IFN-α-2a vs. IFN-α-2a. From Zeuzem et al. [22].

End of Treatment Response	Peg-IFN-α-2a Group		IFN-α-2a Group	
	No. Patients completed treatment = 223		No. Patients completed treatment = 161	
	No. Patients completed follow-up = 206		No. Patients completed follow-up = 154	
	**No**.	**% (95% CI)**	**No**.	**% (95% CI)**

		**Response at week 48**		

Virological	185	69 (63-75)	73	28 (22-33)

Biochemical	123	46 (40-52)	104	39 (33-46)

Both virological and biochemical	109	41 (35-47)	65	25 (20-30)

		**Response at week 72**		

Virological	103	39 (33-45)	50	19 (14-24)

Biochemical	120	45 (39-51)	65	25 (20-30)

Both virological and biochemical	101	38 (32-44)	46	17 (13-23)

IFN = Interferon, Peg-IFN = pegylated-Interferon

### Peg-IFN-α-2a versus Peg-IFN- α-2b

The FDA has approved both pegasys (peg-IFN-α-2a) and PEG-Intron (peg-IFN-α-2b) for use as monotherapies and in combination for the treatment of chronic HCV in patients with compensated liver disease who have not been treated previously. Some of the differences between the two drugs are summarized in Table 2.

**Table 2:** A comparison of Peg-IFN-α-2a and Peg-IFN-α-2b

	Peg-IFN-α-2a	Peg-IFN-α-2b	Remark
**Size**	Large polyethylene glycol moiety	Relatively small polyethylene glycol moiety	Polyethylene glycol moiety in Peg-IFN-α-2a is twice the size of that in Peg-INF-α-2b [9].

**Common Dose**	180 μg/week subcutaneously	1.5 μg/kg/wk subcutaneously	

Efficacy*	Worse	Better	

**Infant and Neonatal use**	Contraindicated	Safe	Peg-IFN-α-2a contains benzyl alcohol [9].

*The results of a randomized trial suggested that, despite its relatively short half-life, Peg-IFN-α-2b results in a greater decrease in HCV RNA than Peg-IFN-α-2a during the first four weeks of monotherapy [25]. However, there was no difference observed between Peg-IFN-α-2b plus ribavirin and Peg-IFN-α-2a plus ribavirin in the treatment of chronic hepatitis C in HIV-infected patients [26]

### Ribavirin

Ribavirin is a synthetic nucleoside antagonist that is active against a broad range of viruses [27,28]. It hinders the initiation and elongation of RNA fragments by inhibiting the synthesis of viral proteins [4,28]. However, the mechanism by which it inhibits HCV RNA in combination therapy with interferon products has not been established [4,11]. Using ribavirin alone as a monotherapy for hepatitis C is not recommended, as other treatments (including ribavirin combined with interferon) are more effective [29-32].

The results of a randomized, double blind, placebo-controlled study conducted to evaluate the role of ribavirin in the treatment of chronic hepatitis C showed that ribavirin monotherapy, given for periods as long as 12 months, is of limited use as a therapy for chronic hepatitis C. In this study, a rapid decrease in serum aminotransferase levels was observed in 54% of patients who were treated with ribavirin. However, only 7% of patients had normal levels of aminotransferase after the discontinuation of ribavirin and there was no change in the serum HCV RNA levels during or after the treatment, proving that the therapy is ineffective [29]. The outcome of a study by Dusheiko et al. indicates that there was no difference between the treatment group and the placebo group in the reduction of HCV RNA levels six months after ribavirin therapy was discontinued. As a result, they concluded that ribavirin was no more effective than a placebo in the treatment of HCV [30].

Also, ribavirin has several adverse side effects that make it less preferable over other treatments [32]. For example, hemolytic anemia is a common consequence of ribavirin use, but a lower, less-effective dose of the drug may be necessary to reverse the reduction of hemoglobin values [9,32]. Other frequent side effects of ribavirin include pruritus, rash, indigestion, loss of appetite, nausea, headache, and fatigue [11,32].

### Pegylated interferon and ribavirin

The highest sustained virologic response (SVR) in patients with hepatitis C has been achieved by a treatment combining peg-IFN -α-2a and ribavirin. This combination therapy has thus become the current standard of care for patients with chronic hepatitis C who have not been treated previously [33].

The findings of a large randomized clinical trial of 1530 patients with chronic hepatitis C show a significantly higher SVR in the peg-IFN-α-2b plus ribavirin group than in the IFN-α-2b plus ribavirin group during initial treatment [34]. This study also established the dose-dependent nature of peg-IFN: a higher dose (1.5 g/kg) of peg-IFN given once weekly in combination with ribavirin is more effective than the lower dose (0.5 g/kg) administered by the same protocol. Patients given the lower dose also showed a response similar to that of the patients who received a standard dose of IFN-α-2b plus ribavirin. Table 3 summarizes the outcomes of the various treatment groups. Adverse effects were similar in both the peg-IFN-α-2b plus ribavirin group and in the IFN-α-2b plus ribavirin group [34].

**Table 3:** Virological responses in the study by Manns et al. [34]

Groups	Overall SVR	SVR at end of follow-up	SVR by genotype		
			**GT 1**	**GT 2/3**	**GT 4/5/6**

Higher-dose peg-IFN + Rib	65% P < 0.001	54% P = 0.01	42%	82%	50%

Lower-dose peg-IFN + Rib	56%, P = 0.41	47% P = 0.73	34%	80%	33%

IFN + Rib	54%	47%	33%	79%	38%

INF = Interferon, Rib = Ribavirin, SVR = sustained virologic response, GT = genotype. *P values are calculated for comparison with IFN + Rib

A randomized, double blind trial with 1311 participants was conducted to assess the efficacy and safety of 24 versus 48 weeks of treatment with peg-IFN-α-2a a plus a low or standard dose of ribavirin [35]. The results showed that the duration of treatment using peg-IFN-α-2a and ribavirin should be determined based on the HCV genotype. According to this study, HCV genotype 1 is best treated by a 48-week regimen of 180 μg/wk of peg-IFN-α-2a and a standard, weight-based dose (1000 or 1200 mg/d) of ribavirin. Another study showed that a low vitamin D serum level is associated with low response rates and severe fibrosis in genotype 1 [36]. Therefore, Vitamin D supplementation may increase sustained response to pegylated interferon plus ribavirin [37]. HCV genotypes 2 and 3 can be treated adequately with a 24-week regimen of 180 μg/wk of peg-IFN-α-2a and a low-dose (800 mg/d) of ribavirin [35].

The response to therapy in HCV patients is heterozygous and, despite increases in SVR rates, treatment outcomes in certain populations with peg-IFN-α and ribavirin are not optimal and may still be improved [35,38]. Early antiviral response to therapy should be monitored to assist in identifying patients who are less likely to achieve SVR and is therefore critical in the management of HCV patients. Rapid virological response (RVR) and complete early virological response (cEVR) are linked with patients who achieve SVR [39]. Identifying both patient- and virus-related baseline and treatment factors that are associated with RVR and cEVR with peg-IFN-α-2a plus ribavirin therapy may help predict a patient's response to treatment, in addition to supplying information that can be used to enhance viral outcomes [39,40]. For example, adjusting baseline and/or treatment factors, such as drug dose, may increase a patient's chance of a successful response to therapy. Moreover, those patients unlikely to reach RVR or cEVR will be recognized early on and this will diminish needless healthcare expenses and side-effects. A retrospective analysis demonstrated that factors including lower serum HCV RNA concentration, absence of cirrhosis, higher ALT quotient, younger age, white non-Latino ethnicity, and daily on-treatment ribavirin dose > 13 mg/kg were independently linked with achieving RVR and cEVR in patients with HCV genotype 1 and treated with peg-IFN-α-2a plus ribavirin for 48 weeks [41]. They recommended that physicians maintain a higher ribavirin dose in patients to increase the likelihood of achieving SVR and tailoring treatment regimens based on an individual's risk profile may be one approach to achieve optimal antiviral response.

### Protease inhibitors

One of the most important determents of response to therapy is the genetic profile of the patient. Patients with a polymorphic mutation on the IL-28B gene for genotype 1 have poor response to treatment. Currently the standard for care is the combination of peg-IFN-α and ribavirin, but only 40-50% of patients infected with HCV genotype 1 have SVR [35]. Retreatment only achieves SVR in 10-20% of patients, but data released at the International Liver Congress shows SVR rates of 40-80% when protease inhibitors are used, even in the presence of the IL-28B genotype [42-44].

### Telaprevir

Telaprevir (VX-950) is a targeted antiviral therapy for hepatitis C. This drug is administered orally and selectively and reversibly inhibits HCV NS3/4A protease, an enzyme required for viral replication. Studies show that VX-950 has a significant antiviral effect on patients with HCV genotype 1, and is well tolerated [45-47]. It has a synergistic antiviral effect when administered with IFN as demonstrated in several studies [47]. Telaprevir was FDA-approved in May, 2011 after the results of the ADVANCE trial [48]. This trial showed that telaprevir combined with standard therapy cured the virus in 75% of patients compared with 44% of patients who were cured on standard therapy alone. Of the almost 60% of telaprevir -treated patients who had undetectable viral levels at weeks 4 and 12 and who were eligible by the terms of the study to receive 24 weeks total of treatment - half the time required for standard treatment - approximately 90% were cured. There were also substantial benefits of telaprevir in subgroups of patients who generally do not respond well to standard therapy. For example, only 25% of African-Americans treated with standard therapy achieved a viral cure compared to 62% of African-American patients who reached a viral cure with the telaprevir regimen. In another group of patients with advanced liver cirrhosis 62% achieved a viral cure with telaprevir versus 33% on standard therapy [48]. These results confirm the findings of the U.S. Phase 2 PROVE 1 study and the European PROVE 2 study [46].

### Boceprevir

Boceprevir, (SCH 503034), is a peptidomimetic ketoamide HCV NS3 protease inhibitor that binds reversibly to the NS3 active site [49]. It has potential as a time-dependent inhibitor of the NS3 protease in cell-free enzyme assays, and has shown robust in vitro activity in the HCV replicon system. The use of SCH 503034 in combination with IFN was more effective than either therapy alone in inhibiting viral replication [50]. Boceprevir has also been shown to minimize the emergence of resistant HCV strains when combined with the inhibitor HCV-796 [49], and was approved by the FDA in May, 2011. The HCV RESPOND-2 study looked at 403 chronic hepatitis C patients with genotype 1 who still had significant levels of the virus after standard treatment of peginterferon and ribavirin. Significantly more patients were cured with boceprevir than with peginterferon and ribavirin [51]. A second study, HACV SPRINT-2, examined patients with chronic HCV genotype 1 infection who had not yet undergone standard treatment, and it was found that there were significantly increased rates of sustained virologic response [52].

Although these direct anti-virals are effective in the treatment of both treatment-naïve HCV patients and those patients who were previously unresponsive to current treatment options, the development of resistant viral variants may be cause for worry. Two studies have found HCV strains resistant to these novel antiviral compounds even for patients who had never been previously exposed to the new antiviral compounds [53-55]. Each new copy of the HCV genome exhibits on average one nucleotide change per replication cycle; this allows the virus to quickly come up with mutations that render it resistant to antiviral drugs. This is a major concern for successful anti-HCV therapy [56].

A significant number of HIV patients are also infected with HCV. With improved survival rates in the HIV population liver disease caused by HCV becomes a serious issue in the health and survival of these patients [57]. HCV patients who are co-infected with HIV have an increased risk of accelerated end stage liver disease and hepatocellular carcinoma than patients infected only with HCV. Therefore treatment should be considered a priority in this group [58]. In October, 2011 the European AIDS Clinical Society (EACS) and the American Association for the Study of Liver Diseases (AASLD) added telaprevir and boceprevir in their guidelines for HIV/HCV co-infected patients [59,60].

There are several other antiviral drugs in different phases of clinical trials. Table 4 summarizes the current drugs that are under development [61].

**Table 4:** A summary of current Hepatitis C drugs based on proteases, polymerase, and other degrading enzymes that are in preclinical development or in clinical trials

Drug Name	**Pharma Co**.	Class	Clinical Trial Phase
TMC435350	Tibotec & Medivir	Protease inhibitor	II

R1626	Roche	Nucleoside polymerase inhibitor	II

DEBIO-025	Debiopharm	Cyclophilin inhibitor	II

Celgosivir	Migenix	α-glucosidase inhibitor	II

BI12202	Boehringer	Protease inhibitor	I

MK-7009	Merck	Protease inhibitor	I

ITMN-191	InterMune & Roche	Protease inhibitor	I

NIM-811	Novartis	Cyclophilin inhibitor	I

R7128	Pharmasset & Roche	Nucleoside polymerase inhibitor	I

PSI-6130	Pharmasset	Nucleoside polymerase inhibitor	I

MK-0608	Merck	Nucleoside polymerase inhibitor	Preclinical

### Pharmacoeconomics of HCV Therapy

Since the treatment of HCV with peg-IFN and ribavirin is expensive, it is necessary to evaluate the cost-effectiveness of the available treatment options. The wholesale price of a 48-week supply of peg-IFN plus ribavirin is about $30,000, while a comparable supply of nonpegylated IFN plus ribavirin costs between $15,000 and $20,000 [9]. A study by Salmon et al. examined the cost-effectiveness and benefits of the current treatments for a chronic hepatitis C infection in asymptomatic and HCV seropositive individuals. The outcome of the study showed that the cost of therapy depends on the genotype and the gender of the patients [62]. Another systematic review of several randomized clinical trials, reports, and meta-analyses reinforced the cost effectiveness of initial combination therapy to prolong life, and improve quality of life [63]. Generally, before beginning treatment, a thorough evaluation of the appropriateness of the therapy is needed, and all decisions regarding the treatment should be made by the patient. Malone et al. compared the cost-effectiveness of treatment with peg-IFN-α-2b plus ribavirin with that of peg-IFN-α-2a in hypothetical cohorts of 100 chronic HCV patients, 75% of whom had a genotype 1 infection [64]. According to the study, the use of peg-IFN-α-2b and ribavirin may be cheaper than peg-IFN-α-2a for the treatment of the hypothetical cohort.

New research has also determined that patients in the United States with HCV are twice as likely not to have health insurance as patients without HCV. Only a third of HCV infected Americans have access to antiviral therapy. The remaining patients are either not candidates for therapy owing to treatment contraindications or are uninsured [65].

### Future treatment options

Despite recent advances in the treatment of chronic hepatitis C, the current treatment options have been ineffective in a significant number of patients, and there is an unmet need for novel, potent, and well-tolerated anti-HCV drugs. Currently, researchers are investigating different treatments for HCV, with the hope of finding more effective and safer therapies; some of the potential candidates are discussed below.

### Cyclophilin inhibitors

Cyclophilins are cyclosporin binding proteins and are classified as a family of peptidyl-prolyl cis-trans isomerases. These enzymes catalyze the cis-trans interconversion of peptide bonds amino-terminal to proline residues, resulting in a conformational change in the protein structure. There are more than 10 subgroups of cyclophilins in mammals [66]. Medications that inhibit these proteins may be an effective treatment against hepatitis C infections. Debio 025 is a cyclophilin B (CyPB) inhibitor taken orally that blocks HCV replication by inhibiting CyPB, a cellular cofactor of the NS5B RNA-dependent RNA polymerase [66]. It also has also the ability to delay or prevent HCV from developing resistance against other antiviral drugs [67]. SCY-635 is a non-immunosuppressive derivative of cyclophilin A that is also taken orally. In vitro studies have shown that this drug exhibits synergistic antiviral activity when administered with INF-α-2b and additive antiviral activity when given with ribavirin [68].

### Immunomodulatory agents

Several toll-like receptor (TLR) agonists are being investigated as possible drugs to stimulate protective antiviral immunity. A phase 1 study of CPG 10101, a synthetic oligodeoxynucleotide, demonstrated that it acts as an immunomodulator and decreases HCV RNA levels [69]. Isatoribine is a TLR7 agonist that produces a significant dose-dependent drop in plasma HCV RNA without major side effects [70].

### Thiazolides

Studies have noted that thiazolides, such as nitazoxanide and tizoxanide, are potent inhibitors of HCV replication, and suggest that thiazolides may enhance current or future anti-hepatitis treatments [71-73].

### Vaccines

At the present, there is not an effective vaccine against HCV [5], and efforts to develop a vaccine have been met with many difficulties [1,3,5]. The genetic diversity among the HCV genotypes makes it challenging to create a single vaccine effective against all types of the virus [1,3,5], and its fast mutation rate further complicates the issue [4]. Nevertheless, findings from various studies show promising advances in the development of new immunotherapy treatments against HCV.

### HCV-LP

An animal study showed that immunization with hepatitis C virus-like particles (HCV-LPs) results in the induction of certain HCV-specific cellular immune responses that can control a hepatitis C virus infection in chimpanzees [74]. Another study in baboons found that after a course of treatment with HCV-LPs all the animals exhibited an HCV-specific immune response and developed antibodies to HCV proteins [75].

### rDNA and adenovirus vaccines

The induction of long-term HCV-specific antibody and T-cell responses was demonstrated in chimpanzees as a result of immunization with recombinant DNA and adenovirus vaccines expressing HCV core, E1E2, and NS3-5 genes; a substantial reduction in the viral load was also observed [76].

### Dendritic cells transduced with an adenovirus encoding NS3 protein

This is a new immunization approach using dendritic cells, the most potent antigen presenting cells, loaded with viral antigens to induce antiviral immunity [77,78]. Other immunomodulatory agents and vaccines are currently in clinical trials [79].

### Prevention and control of HCV

The Centers for Disease Control and Prevention (CDC) recommend implementing primary and secondary prevention activities in order to control the spread of HCV [80]. Primary prevention activities include virus inactivation in blood products, testing organ and tissue donors, and providing counseling on risk reduction [5,80]. These activities aim to reduce the risk of contracting HCV through the main transmission routes, including the use of contaminated drug needles, engaging in sexual intercourse with infected persons, and percutaneous exposure to infected blood in the health care and other (e.g. tattooing and body piercing) fields [80]. Secondary prevention activities, such as counseling, identifying and testing people at risk, and treating infected patients, are intended to reduce the risks for liver and other chronic diseases in those patients infected with HCV [5,80]. Providing education for healthcare professionals and the general public, along with surveillance and research to monitor disease trends are also parts of the comprehensive strategy recommended by the CDC [80].

## Conclusions

Although great advances have been made in the development of immunotherapies for HCV, this field is challenged by the lack of adequate knowledge of the virus and its interaction with the host cells. One of the reasons for these difficulties is the absence of a simple animal model for the disease, as only chimpanzees can be used for this purpose. The results of several studies on the development of effective vaccines and powerful anti-virals show that more and highly effective protective treatment options will be available in the near future. The current standard for care, according to the American Association for the Study of Liver Diseases and European guidelines, is a combination of pegylated IFN and ribavirin with or without a protease inhibitor in patients with genotype 1 infection, taking note that these should be prescribed with caution due to the discovery of resistant strains. Other factors that may influence response to therapy, such as HDL levels, serum vitamin D levels, IL-28B genotype, viral load and genotype, and RVR/cEVR, should be taken into consideration. Implementing various prevention and control strategies, promptly identifying and treating acutely infected individuals, creating public awareness of the disease, and continuing HCV research should all be of the highest importance.

### Recently published phase IIa data for successful HCV genotype 1 treatment with two antiviral agents for patients who previously did not respond to peginterferon and ribavirin therapy

Results reported since peer review of this article showed a SVR was achieved in HCV genotype 1 patients treated with only two antiviral agents (asunaprevir and daclatasvir), and that a high rate of SVR was obtained when these two agents were combined with peginterferon alfa-2a and ribavirin, at 12 weeks after treatment. Notably, the success rate was 100% in patients receiving the combination therapy of the two antivirals plus peginterferon alfa-2a and ribavirin [81].

## Abbreviations

ALT: Alanine aminotransferase; AASLD: American Association for the Study of Liver Disease; cEVR: Complete early virological response; CyPB: Cyclophilin B; EACS: European AIDS Clinical Society; HCV: Hepatitis C virus; HCV-LPs: Hepatitis C virus-like particles; IFN: Interferon; peg-IFN: Pegylated interferon; RVR: Rapid virological response; SVR: Sustained virologic response; TLR: Toll-like receptor.

## Competing interests

The authors declare that they have no competing interests.

## Authors' contributions

WA performed literature searches and drafted the manuscript. SM and GS participated in editing the manuscript and in particular SM added text based on the reviewers' comments. SAM participated in drafting the outline and editing of the manuscript. All authors read and approved the final manuscript.
